# Cameron County Medical Society

**Published:** 1886-02

**Authors:** Vol Reed

**Affiliations:** Sec’y.; Cameron, Texas


CAMERON, TEXAS, Jan. 7, 1886.

EDITOR DANIEL'S TEXAS MEDICAL JOURNAL:

THE physicians of Milam county, Texas, met on the 5th inst., in the town of Cameron, and organized a County Medical Society. Dr. W. R. Kennard, of Rockdale, was elected president; Dr. W. F. Sharp, of Davilla, and Dr. D. Monroe, of Cameron, vice-president; Dr. Vol Reed, of Cameron, secretary and treasury. A committee on constitution and by-laws was appointed consisting of Drs. J. E. Cofer, C. W. Macune and Vol Reed, of Cameron; and on motion of Dr. E. J. Powell, Drs. A. C. Walker and W. R. Kennard, of Rockdale, were added to the committee. A committee on credentials was appointed by the chair, consisting of Drs. A. C. Walker, W. N. Green and E. J. Powell. Dr. A. C. Walker was chosen to read an essay at our next meeting. On motion of Dr. C. W. Macoune, the secretary was ordered to furnished a condensed report of the proceedings to DANIEL'S MEDICAL JOURNAL. Adjourned to meet again on the 19th inst. to effect a more permanent organization. Yours respectfully, VOL REED, Sec'y.

